# Effect of Curing Rate on the Microstructure and Macroscopic Properties of Epoxy Fiberglass Composites

**DOI:** 10.3390/polym10020125

**Published:** 2018-01-27

**Authors:** Ammar Patel, Oleksandr Kravchenko, Ica Manas-Zloczower

**Affiliations:** 1 Department of Macromolecular Science and Engineering, Case Western Reserve University, Cleveland, OH 44106, USA; aap89@case.edu; 2 Department of Mechanical and Aerospace Engineering, Old Dominion University, Norfolk, VA 23529, USA; okravche@odu.edu

**Keywords:** curing rate, epoxy microstructure, fatigue, composites, critical gel

## Abstract

Curing rates of an epoxy amine system were varied via different curing cycles, and glass-fiber epoxy composites were prepared using the same protocol, with the aim of investigating the correlation between microstructure and composite properties. It was found that the fast curing cycle resulted in a non-homogenous network, with a larger percentage of a softer phase. Homogenized composite properties, namely storage modulus and quasi-static intra-laminar shear strength, remained unaffected by the change in resin microstructure. However, fatigue tests revealed a significant reduction in fatigue life for composites cured at fast curing rates, while composites with curing cycles that allowed a pre-cure until the critical gel point, were unaffected by the rate of reaction. This result was explained by the increased role of epoxy microstructure on damage initiation and propagation in the matrix during fatigue life. Therefore, local non-homogeneities in the epoxy matrix, corresponding to regions with variable crosslink density, can play a significant role in limiting the fatigue life of composites and must be considered in the manufacturing of large scale components, where temperature gradients and significant exotherms are expected.

## 1. Introduction

High performance thermoset composites are ubiquitous all around us. They are selected due to their light weight, high glass transition temperature, high strength and great chemical resistance [1,2,3]. The majority of the strength in a structural composite comes from the fiber being used for reinforcement, while the resin acts as a binder for the fiber mat holding the mat together [4,5]. Epoxies mixed with a curing agent or hardener represent a large percentage of thermoset resins, largely due to their excellent wettability and strong adhesion to the fiber. Epoxy composites are currently used in the manufacture of boat hulls, wind turbine blades, aircrafts and cars, to serve as structural materials. Despite the strength of the composite, stemming from the fiber reinforcement, the matrix has a large role to play in the failure of composites. The primary role of the matrix is to provide the stress transfer between the fibers. Therefore, failure of the composite usually occurs through matrix cracking, interface debonding, delamination or fiber breakage [6,7,8]. The microscopic damage in the matrix deteriorates the composite’s mechanical properties and eventually results in coalescence of microscopic cracks and formation of transverse ply cracks and delaminations, which can lead to catastrophic failure of the composite. Thus, it is essential to study the microstructure of the matrix and understand how local non-homogeneities in the epoxy can affect the overall performance of the composite.

On a molecular scale, the epoxy amine reaction involves the opening up of the epoxide ring by reaction with an amine hydrogen [9,10,11,12,13]. The initial cure is largely dominated by the primary amine reaction taking place since the secondary amines are sterically hindered [14]. A post cure is thus required to ensure complete conversion of the secondary amines. On a macro scale, these reactions manifest themselves as the epoxy resin and curing agent mixture transforming from a fairly viscous liquid to a gel, through the formation of a three dimensional network, and finally, to a cross-linked intractable solid [15,16]. The onset of gelation was shown to be important for predicting residual stress development in composites. Previous studies have shown that residual stress build up in composites occurs past the gel point and is due to cure induced shrinkages in the epoxy matrix [17,18]. Cure rates past the gel point were shown to significantly affect residual deformation in composite laminates [19,20], while residual stresses alone can be significant enough to result in processing-induced damage in composites after cure. However, the method by which the cure conditions prior to gelation affect composite mechanical performance still remains not fully understood. Several studies have examined the microstructure of the epoxy network to get a better idea of the way gelation occurs [21,22,23,24]. In each of these studies, a two-phase structure was observed, consisting of a highly cross-linked, high density nodular phase, interspersed with a low density, softer phase. Small highly cross-linked microgel particles, in the order of ~10 nm, were the characteristic feature of the nodular phase, with larger nodules formed by the aggregation of these microgels. These investigative studies provided a model for gelation wherein:

1. Microgel particles, made up of uniformly cross-linked networks, nucleate and grow from the reacting polymeric network.
2. These microgels begin to coalesce, to form less coherent clusters, which continue to interact with other microgel clusters.
3. At gelation, the microgel clusters pack together to form a non-uniform, incoherent gel network with the partially reacted networks having a lower cross-link density, forming the dispersed phase.

Previous studies have manipulated the microstructure of the epoxy network by changing the stoichiometry of the epoxy amine reaction [22]. It was found that increasing the epoxy or amine content decreased the connectivity of the microgel clusters, resulting in a higher fraction of a dispersed phase. Studies on varying the stoichiometry of the epoxy amine reaction show either a decrease in mechanical properties or a decrease in the glass transition temperature (*T*_g_), due to the presence of excess amine or epoxy [22,25,26]. Therefore, this does not present an effective way of tuning the microstructure of the epoxy, especially in the case of structural composites where high strength and *T*_g_ are required. In this work, we propose changing the microstructure by modifying the curing rate of the epoxy amine reaction. There are two major questions that need to be answered: What is the effect of increasing the reaction rate up to gelation? Can the reaction proceed so quickly that the microgels are unable to coalesce completely?

To the best of our knowledge, varying the curing temperatures, and thus, the curing rates, to study the microstructure of the resulting matrix, and observing the effect of this change on the composite properties, has not been studied. Increasing the curing rate, leading to quicker cycle times, also has the benefit of expediting composite manufacturing, which is of practical importance to industry. This work involves studying the effect of three different curing cycles on the evolution of the epoxy network and the implication it has on the final composite properties.

## 2. Experimental

### 2.1. Materials

Lab grade Diglycidyl ether of Bisphenol A (DGEBA) resin and the curing agent, isophorone diamine (IPD) are a product of Sigma–Aldrich^®^ (St. Louis, MO, USA). Bi-directional glass fiber mats were procured from Fiberglast^®^ (Brookville, OH, USA).

The epoxy resin and curing agent were mixed together using a glass rod for 5 min. The Epoxide Equivalent Weight (EEW) of DGEBA was 170 g/equivalent, obtained directly from the manufacturer mentioned above. The curing agent was added such that the ratio between the EEW and the Amine Hydrogen Equivalent Weight (AHEW) was 1:1. The Amine Hydrogen Equivalent Weight was calculated by the following equation.

(1) $$\mathrm{Amine}\;\mathrm{hydrogen}\;\mathrm{equivalent}\;\mathrm{weight}\;=\;\frac{\mathrm{Molecular}\;\mathrm{weight}\;\mathrm{of}\;\mathrm{Amine}}{\mathrm{number}\;\mathrm{of}\;\mathrm{active}\;\mathrm{hydrogens}}=\frac{170.30}{4}=42.575$$

The amount of amine added in parts per hundred (phr) was thus found by

(2) $$phr=\frac{AHEW}{EEW}\;\times \;100\;=\;\frac{42.575}{170.3}=25\;g\;\mathrm{per}\;\mathrm{hundred}\;\mathrm{grams}\;\mathrm{of}\;\mathrm{epoxy}$$

The composites were prepared by the hand layup method. Five layers of glass fiber mats were each impregnated with the resin curing agent mixture and stacked one on top of the other. Three different curing rates were used to cure the composite samples. After impregnation, one composite was allowed to cure at room temperature (RT) for 12 h, following which it was hot pressed, at a pressure of 2 metric tons, at 70 °C for 6 h, and then 140 °C for 6 h. This sample will be referred to as the “RT sample” in the rest of the manuscript. Another impregnated composite, termed the “70 sample”, was hot pressed, with a pressure of 2 metric tons, at 70 °C for 6 h, and 140 °C for 6 h. A third composite, labeled the “140 sample”, was directly hot pressed, at 140 °C, at a pressure of 2 metric tons, for 6 h following impregnation. The fiber mats used per composite were weighed prior to impregnation and the composite was weighed again post-curing, to obtain the fiber to resin weight ratio. For all the 3 curing cycles, the weight ratio of the composite was 65 parts fiber and 35 parts epoxy. In order to study the microstructure of the epoxy network, control samples without any glass fiber mats were also prepared. The epoxy resin and hardener were mixed together, and the mixture was poured into rectangular shaped molds and cured via the 3 curing cycles. Similar to the composite samples, these were labelled as “RT sample”, “70 sample” and “140 samples”, respectively.

### 2.2. Instrumental Methods

#### 2.2.1. Soxhlet Extraction

Weighted samples obtained from the 3 different curing cycles were refluxed in Tetrahydrofuran (THF) for 72 h, following which the samples were dried to remove all solvent and weighed again to measure the difference in weight. The experiment was repeated from different samples at least twice for reproducibility. It was expected that all unreacted or partially reacted monomers of the epoxy amine reaction would be washed away during the THF reflux.

#### 2.2.2. Differential Scanning Calorimetry (DSC) 

DSC was performed on test samples using a TA Instrument DSC Q100 (New Castle, DE, USA) under nitrogen flow, using aluminum hermetic pans obtained from TA Instruments. Samples were subjected to a temperature ramp, from −60 to 200 °C, at a ramp rate of 3 °C/min. Samples were then equilibrated to −60 °C and ramped once more to 200 °C, at a rate of 3 °C/min. The glass transition temperature (*T*_g_) of the samples was obtained as the midpoint of the drop in heat capacity during the heating cycle. Experiments were repeated 2–3 times to ensure reproducibility.

#### 2.2.3. Atomic Force Microscopy (AFM)

Phase images to probe the microstructure of the epoxy samples were obtained using the Veeco Dimension 3100 Atomic Force Microscope (Bruker, Billerica, MA, USA) in Tapping mode with a Si-Carbide tip with a length of 125 mm, a spring constant of 40 Nm^−1^ and a resonant frequency of 372 KHz. Samples were microtomed, using a diamond knife, prior to imaging. Scan sizes of 250 nm and 1 μm were probed at a scan rate of 0.513 Hz. The contrast in phase images is affected by the viscoelastic behavior of the matrix, leading to brighter areas having a higher cross-link density. The images obtained from the AFM were analyzed using ImageJ to find the percentage of bright and dark areas. During this analysis, care was taken to neglect those areas observed in the phase images that were clearly an artifact derived from the valleys and ridges obtained from the height imaging. The maximum surface roughness through the entire scan size did not exceed 35 nm. The thickness of these samples was ~1.5 mm.

#### 2.2.4. Thermal Gravimetric Analysis (TGA)

TGA was performed on test samples using TA Instruments Q500 under nitrogen flow with a ramp rate of 10 °C/min until 600 °C. It is to be mentioned here that due to difficulties in cutting the glass fiber mat, the small amount of sample used primarily consists of the epoxy network and thus, does not reflect the fiber to resin ratio used in the composite.

#### 2.2.5. Dynamic Mechanical Analysis (DMA)

TA instruments Q800 DMA, operating in dual cantilever mode, with an amplitude of 10 μm at a frequency of 1 Hz, was used to determine the Storage Modulus (*E*’). The glass transition temperature was obtained through the peak of the tan delta curve. Samples were run from 40 to 200 °C, at a ramp rate of 3 °C/min. Experiments were repeated 2–3 times for reproducibility.

#### 2.2.6. Static and Dynamic Flexural Testing

Three-point bending tests were performed on the composite samples in static and fatigue modes. The static test was conducted on an Instron Tensile tester (Instron, Norwood, MA, USA) with a load cell of 5 kN at a speed of 2 mm/min and a span length of 50 mm. Dynamic three-point bending tests were performed on a servo-hydraulic test machine (MTS Model 20 kip, Eden Prairie, MN, USA) at the same span length, a frequency of 3 Hz of the loading sinusoidal wave and at a load that was 80% of the maximum load used to break the strongest samples determined from the static load test. The fatigue testing was conducted under load controlled mode with a load ratio, defined as the ratio between the minimum to maximum load, of *R* = 0.2. The number of cycles to failure was recorded and at least three specimens were tested for each sample, to ensure reproducibility.

## 3. Results and Discussion

### 3.1. Probing the Microstructure

The epoxy microstructure exposed to three different curing cycles was probed using the control samples without any glass fiber mats. Multiple pieces of the differently cured epoxies were subjected to Soxhlet extraction in THF. It was expected that a fully cured epoxy network would swell when exposed to THF but not dissolve. The unreacted parts of the network, i.e., the monomers or oligomers that had not cross-linked, would be washed away with the THF, observable by a weight change. A sample of the results for the Soxhlet extraction can be seen in Table 1. Since the initial sample weight changed when repeating the experiment, only the percentage change values reflect multiple tries.

As can be seen in Table 1, the two samples (RT sample and 70 sample) that were allowed to gel completely before the post cure show almost no change in weight after extraction. Thus, the network was less homogenous in the 140 sample, since the microgels were not allowed to coalesce completely during the gelling stage.

**Figure 1:**
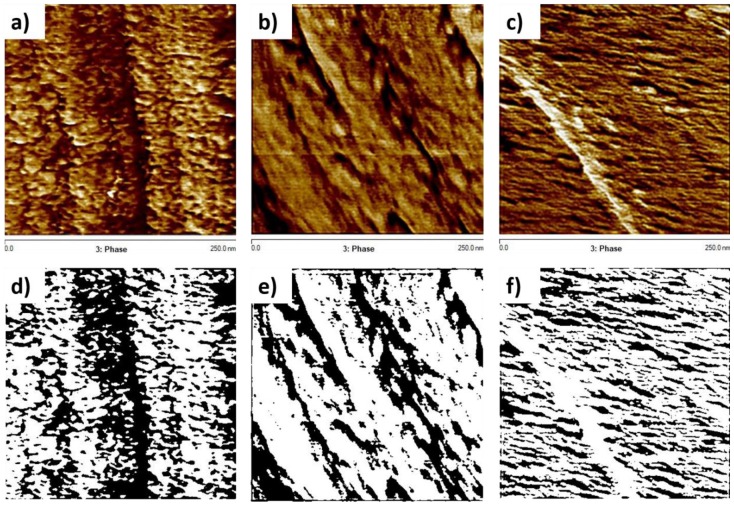
Phase images in tapping mode for (**a**) RT sample (**b**) 70 sample (**c**) 140 sample; figures (**d**–**f**) represent the threshold black and white images used to measure the percentage of bright and dark areas.

AFM results show no significant differences between the samples on the 1 μm scale via the AFM, as can be seen in Supplementary Materials Figure S1. There were no nodular structures of 10–70 μm observed, as mentioned in other works [23,24]. At a scale of 250 nm, however, as seen in Figure 1, the inhomogeneous structure of the 140 sample is readily observable. The height images for all these samples can be seen in Supplementary Materials Figure S2. The fast cure of the 140 °C sample is expected to leave larger areas of partially reacted network that manifest themselves as a softer phase, with reduced mechanical properties, when compared with an otherwise hard epoxy network. These areas are regions of low cross-link density. The 140 sample is regularly interspersed with a softer phase material, indicating microgel clusters, meaning less connectivity between the highly cross-linked clusters. The RT sample, when compared to the 140 sample, shows a more homogenous network on either side of the height artifact. Significant dark spots in the 70 sample are mere artifacts, resulting from the differences in height. The 70 sample shows a similar structure to the RT in the bright spot dominated regions, with small amounts of a softer phase. It is interesting to note that the 140 sample has a similar structure to non-stoichiometric epoxy amine networks observed in other works [22], reinforcing the hypothesis that the fast cure in the 140 sample leaves portions of the system partially cured and thus, soft. Measuring of the relative areas of the bright and dark spots, i.e., the harder and softer phases of the epoxy network, respectively revealed that the 140 sample has a significantly higher percentage of softer phase regions when compared to the other samples (Supplementary Materials Table S1). The RT sample has ~15% of a softer phase region, whereas the 140 sample has ~31% of a softer phase region.

**Figure 2:**
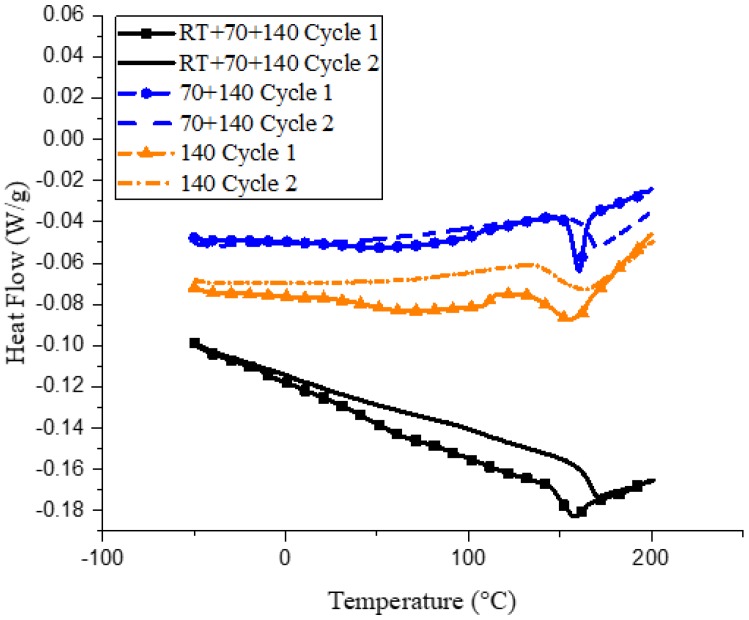
Heat capacity curves for samples cured under the three curing cycles.

Differences in curing via the three cycles can also be observed by looking at the changes in glass transition via the DSC, as can be seen in Figure 2.

All three samples, when subjected to a second curing cycle, show an increase in glass transition temperature. This is due to some amount of residual cure remaining during sample preparation, as is evident from the exothermic peaks observed immediately after *T*_g_. For the 140 sample, a broad dip is observed in the 100–150 °C range, indicating a glassy to rubbery transition for the partially reacted oligomers in the network. This is apparent when comparing the *T*_g_ values of the three samples from the initial curing cycle, wherein the RT and 70 samples show similar *T*_g_ values of ~155 °C, whereas the 140 sample, due to the presence of these partially cured oligomers, has a lower *T*_g_ of 143 °C. However, the curing reaction for these oligomers is expected to persist, provided enough thermal activation is provided. This is exactly what is observed during the second curing cycle. During the second heating cycle, the bump observed in the 140 sample disappears, indicating that the oligomers have reacted. The *T*_g_ of the RT and 70 sample increase to similar temperatures of 165 °C during the second heating cycle, however, the *T*_g_ of the 140 cycle increases only up to 150 °C. Previous work [27] has noted that *T*_g_ is an exponential function of the degree of cure, indicating that close to full cure, a small increase in the degree of conversion causes a big increase in the *T*_g_. This is similar to what is observed in the present DSC data. Within a particular microgel, the epoxy could be completely cured, but total coalescence of the individual microgels is essential for a complete degree of cure, which seems to be lacking in the 140 sample. The cross-linking reaction is still observed at the end of the second curing cycle for all samples because the reaction was not completed during the first cycle before the samples were cooled down, leading to vitrification.

Based on the microstructure, the 140 sample showed a distinct difference when compared to the RT and 70 samples. A closer look at the degree of conversions in the pre-curing stages of these RT and 70 samples provided an explanation for this behavior. As mentioned earlier, the *T*_g_ of the epoxy network increases as the curing reaction progresses [27] and if the *T*_g_ evolves to the temperature of the reaction, the epoxy transforms into a glass, leading to vitrification [28,29,30]. Also, gelation increases the viscosity by close to three orders of magnitude [31], after which the diffusion of the polymeric chains slows down, decreasing reaction kinetics [32]. Using an equation derived previously [27], the degree of conversion of the epoxy reaction can be obtained from the temperature at which the reaction proceeds (T), assuming enough time is given for the *T*_g_ of the network to reach the temperature of reaction.

(3) $$\frac{T-Tg_{0}}{Tg_{\infty }-Tg_{0}}=\frac{\lambda \alpha }{1-\left(1-\lambda \right)\alpha }$$

Here, $Tg_{0}$ is the glass transition temperature of the epoxy monomer, which, in the case of DGEBA, is −25 °C; $Tg_{\infty }$ is the glass transition temperature of the completely cured epoxy network, which was found to be 165 °C from DMA; α is the degree of conversion; and λ is a derived term that can be approximated to $\frac{Tg_{0}}{Tg_{\infty }}$ (both temperatures in Kelvin), which equates to 0.566. Both the RT and the 70 sample are given enough time to cure at 70 °C, which, when substituted into Equation (3), give a value of the degree of conversion of 0.63. According to the Flory Stockmayer theory, a bifunctional epoxy and a tetrafunctional amine, when mixed in a stoichiometric ratio, critically gel at

(4) $$\alpha_{gel}={\left(\frac{1}{\left(f_{a}-1\right)\left(f_{b}-1\right)}\right)}^{0.5}\to \alpha_{gel}={\left(\frac{1}{\left(2-1\right)\left(4-1\right)}\right)}^{0.5}\to \alpha_{gel}=0.577$$

This value is very similar to that obtained for the RT and 70 samples. Thus, enough time is given for the microgels to diffuse and coalesce to reach their critical gel point and stabilize. The 140 sample, on the other hand, cures so quickly with the rise in reaction temperature, and thus, reaction rate, that even though microgel clusters form, the reaction medium has become viscous enough to prevent complete coalescing of these microgels. This leaves a large amount of partially unreacted chains in the network, exhibiting a pseudo-off stoichiometric network structure.

### 3.2. Composite Characterization

**Figure 3:**
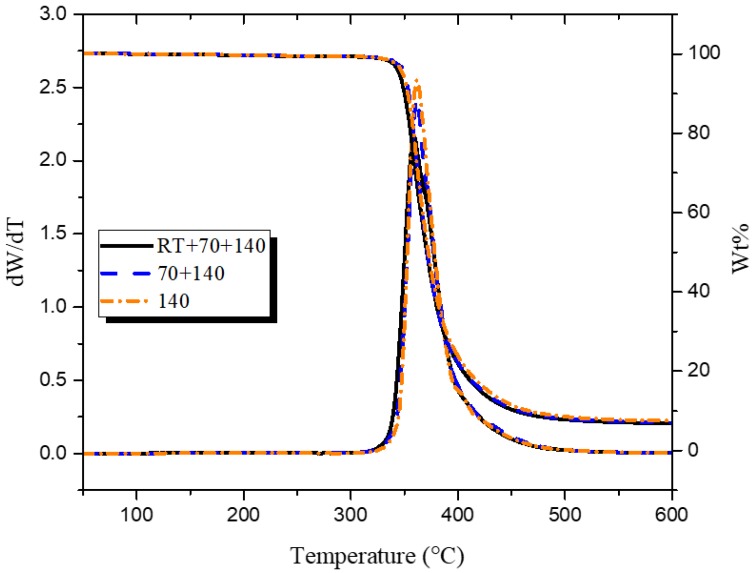
Thermal decomposition of epoxy glass-fiber composites.

After the matrix was characterized, it was important to investigate how the observed changes in the microstructure correlate with macroscopic properties of the composite. The thermal stability of three types of sample was studied by TGA. Weight loss curves as a function of temperature show a one-step degradation process, as can be seen in Figure 3. The onset of thermal degradation at 5 weight percentage loss (*T*_d5%_) and the peak decomposition temperature (*T*_peak_), shown in Supplementary Materials Table S2, were used to observe any differences in thermal decomposition. Values for *T*_d5%_ varied between 345 to 350 °C for all samples, while the difference between the *T*_peak_ values was minimal. Consequently, it is apparent that considered changes in microstructure, due to the differences in heating rates, did not translate into changes in the decomposition temperature of the composite.

A dynamic mechanical analysis, obtained from DMA and static flexural testing, revealed that macroscopic composite properties were not affected by post-curing conditions. As can be seen in Table 2, the macroscopic properties of storage modulus, above and below the glass transition temperature (Supplementary Materials Figure S3), flexural modulus (calculated from the linear region of the load-displacement curve), flexural strength (measured as the stress required for failure on the mid span of the outermost layer of the composite,) and strain at break, do not change significantly between samples (Supplementary Materials Figures S4–S6). Flexural stress was calculated using Equation (5)

(5) $$\mathrm{Flexural}\;\mathrm{stress}\;=\;\frac{3FL}{2bd^{2}}$$

where F is the force applied, L is the span length (50 mm), b is the width of the sample and d is the thickness of the sample.

Similar values for fiberglass epoxy composites have been observed in previous works [33]. This behavior was expected, since the strength of the fibers will be dominant during quasi-static loading [34]. It is known that the presence of the hydroxyl groups on the glass fiber surface can accelerate the cure behavior of the epoxy amine reaction. It was hypothesized that this acceleration might be playing a role in changing the microstructure, leading to similar macroscopic properties. Thus, control samples used to probe the microstructure were also observed on the DMA under the same conditions. The results which can be seen in Supplementary Materials Table S3 show that the changes in curing cycle still do not affect the macroscopic properties. The values for storage modulus, above and below the glass transition, for the neat epoxy samples, are similar irrespective of the curing cycle.

**Figure 4:**
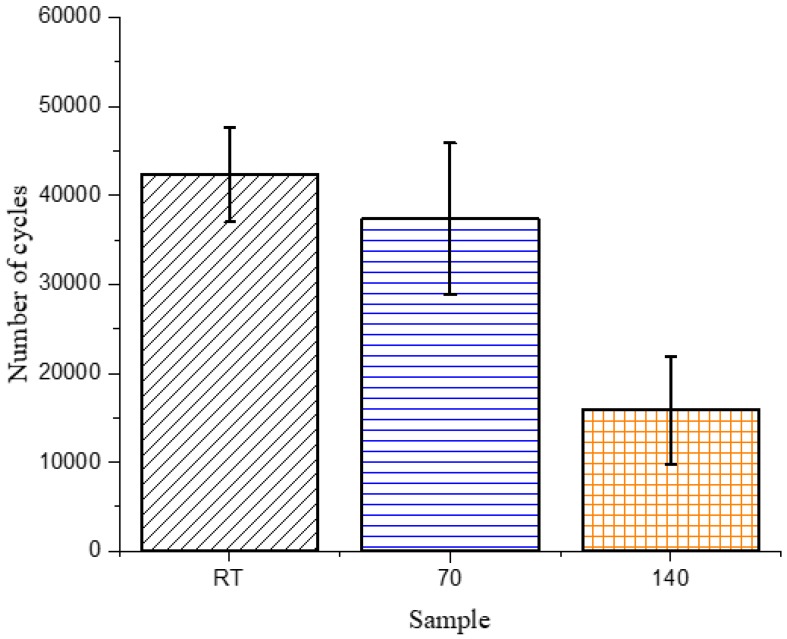
Number of cycles to failure in three-point bending fatigue tests for composite samples.

The only difference that could be observed was in the tan delta peak height (Supplementary Materials Figure S7), which showed that the 140 sample had a lower peak height, when compared to the similar peak heights observed for the RT and 70 samples. Since tan delta is qualitatively known to reflect damping or relaxation of the matrix, fatigue testing was done, to determine if the microstructural changes developed during curing would affect the wear and relaxation of the composites. As mentioned previously, the common failure modes in composites are matrix cracking, interface debonding and delamination [6,7,8], which develop from coalescence of microscopic cracks throughout the fatigue life of a composite, thus, changes in the microstructure of the matrix were expected to have noticeable differences during cyclic testing under subcritical loads. The maximum load during the cyclic test was kept constant for the three types of composites and was selected as 80% of the maximum flexural force required to fail the RT sample (88.2 N). The number of cycles required for the 140 sample to fail was significantly lower than those required for the RT and 70 samples, as shown in Figure 4.

The change in the matrix microstructure, as depicted in the tan delta peak, is exactly reflective of the results obtained from the fatigue testing. The larger region of softer phase in the 140 sample would act as a stress concentration site and break prematurely, leading to early failure in those samples. The 70 and RT samples, having similar microstructures and tan delta peak heights, undergo a similar number of cycles to fail.

## 4. Conclusions

The epoxy samples were cured under different conditions, to obtain variations in their microstructures. It was found that those samples that were allowed to gel completely before post-curing showed similar microstructures and mechanical properties, whereas the sample that was cured significantly faster showed a larger region of a softer (lower crosslink density) phase, observable in the Soxhlet extraction, DSC and AFM. When using these curing cycles in epoxy glass fiber composites, it was found that static flexural tests, storage modulus and thermal decomposition remained unchanged, irrespective of microstructure. This result was explained by the fact that the corresponding properties are governed by the homogenized mechanical response of the composite, rather than the local non-homogeneous microstructure in epoxy matrix. The presence of microstructural variance in the matrix was found to play a significant role under fatigue loading, where the composite response is determined by the local “hot” spots in the microstructure, rather than the overall homogenized properties of the composite. The presence of soft sub-microscopic regions of epoxy with lower cross-link density played the role of weak links in the material, which was evident under fatigue loading conditions, where material degradation was gradual, but not in quasi-static loading. Consequently, composites manufactured with faster cure rates, and as a result containing greater volume content of the soft epoxy phase, showed lower fatigue life. However, if given enough time to critically gel, samples showed similar microstructures and thus, similar fatigue properties, irrespective of the rate of cure prior to gelation. Curing times can be expedited by pre-curing the composite at a temperature which vitrifies the network at the critical gel point degree of conversion, without having any consequences on the properties, as is apparent in the RT and 70 samples.

The present result has important practical significance for predicting the fatigue life of composite structures, which normally show a distribution of the cure rate profiles, as a result of the heat transfer and heat generated from the exothermic reaction during epoxy cure. Therefore, the varying cure rates would result in locally different microscopic compositions of the hard-soft phases in the epoxy matrix, and can be expected to affect the fatigue life of composite structure. Thus, the consideration of the cure state prior to gelation is an important aspect of predicting fatigue life of composites, as is the consideration of residual stresses, which are largely affected by the cure reaction past the gel point.

## Figures and Tables

**Table 1:** Soxhlet extraction of samples cured under the three curing cycles.

Curing Cycle	Weight before Drying (mg)	Weight after Drying (mg)	Percent Change in Weight (%)
Room temperature (RT) sample	258.6	258.4	0.077 ± 0.002
70 sample	176.7	176.2	0.275 ± 0.024
140 sample	294.8	286.1	2.886 ± 0.212

**Table 2:** Storage modulus, glass transition temperature (*T*_g_) and flexural properties of epoxy glass-fiber composites.

Sample	Storage Modulus at 40 °C (GPa)	Storage Modulus at 200 °C (GPa)	*T*_g_ (°C)	Flexural Modulus at 25 °C (GPa)	Flexural Strength at 25 °C (MPa)	Strain at Break (%)
RT + 70 + 140	13.8 ± 0.1	3.8 ± 0.3	165	20.6 ± 1.4	354 ± 17.7	1.9 ± 0.1
70 + 140	13.4 ± 0.1	3.7 ± 0.2	164	20.1 ± 0.6	350.4 ± 35.1	2 ± 0.2
140	12.5 ± 0.3	3.6 ± 0.4	165	20.2 ± 1.2	343.1 ± 41.0	2.1 ± 0.2

## Supplementary Materials

The following are available online at http://www.mdpi.com/2073-4360/10/2/125/s1, Figure S1: Phase images of (a) RT sample (b) 70 sample (c) 140 sample at 1μm scale size, Figure S2: Height images of a) RT sample b) 70 sample c) 140 sample at 250nm scale size, Table S1: Percentage area of softer phase interspersed between the hard epoxy network, Table S2: Onset of thermal degradation at 5 weight % loss and peak degradation temperature for the samples cured at different rates, Figure S3: DMA curves for epoxy glass fiber composites , Figure S4: Flexural 3 point bending curves for RT sample, Figure S5: Flexural 3 point bending curves for 70 sample, Figure S6: Flexural 3 point bending curves for 140 sample, Table S3: Dynamic Mechanical Analysis of sample without glass fiber mats, Figure S7: Tan delta vs temperature for the 3 curing cycles without glass fiber mats.

## References

[1] Fan Z., Santare M.H., Advani S.G. (2008). Interlaminar shear strength of glass fiber reinforced epoxy composites enhanced with multi-walled carbon nanotubes. Compos. Part A Appl. Sci. Manuf..

[2] Brøndsted P., Lilholt H., Lystrup A. (2005). Composite Materials for Wind Power Turbine Blades. Annu. Rev. Mater. Res..

[3] Pilato L.A., Michno M.J. (1994). Phenolic Resins: A century of progress. Advanced Composite Materials.

[4] Thwe M.M., Liao K. (2002). Effects of environmental aging on the mechanical properties of bamboo-glass fiber reinforced polymer matrix hybrid composites. Compos. Part A Appl. Sci. Manuf..

[5] Latha P.S., Rao M.V., Kumar V.K., Raghavendra G., Ojha S., inala R. (2016). Evaluation of mechanical and tribological properties of bamboo–glass hybrid fiber reinforced polymer composite. J. Ind. Text..

[6] Akinyede O., Mohan R., Kelkar A., Sankar J. (2009). Static and Fatigue Behavior of Epoxy/Fiberglass Composites Hybridized with Alumina Nanoparticles. J. Compos. Mater..

[7] Birger S., Moshonov A., Kenig S. (1989). Failure mechanisms of graphite-fabric epoxy composites subjected to flexural loading. Composites.

[8] Lucas J.P. (1992). Delamination fracture: Effect of fiber orientation on fracture of a continuous fiber composite laminate. Eng. Fract. Mech..

[9] Patel A., Maiorana A., Yue L., Gross R.A., Manas-Zloczower I. (2016). Curing Kinetics of Biobased Epoxies for Tailored Applications. Macromolecules.

[10] Mijovic J., Fishbain A., Wijaya J. (1992). Mechanistic modeling of epoxy-amine kinetics. 2. Comparison of kinetics in thermal and microwave fields. Macromolecules.

[11] Pascault J.P., Moschiar S.M., Williams R.J.J. (1990). Buildup of Epoxycycloaliphatic Amine Networks. Kinetics, Vitrification, and Gelation. Macromelecules.

[12] Mijovic J., Fishbain A., Wijaya J. (1992). Mechanistic modeling of epoxy-amine kinetics. 1. Model compound study. Macromolecules.

[13] Mijovic J., Wijayat J. (1994). Reaction Kinetics of Epoxy/Amine Model Systems. The Effect of Electrophilicity of Amine Molecule. Macromolecules.

[14] Pollard M., Kardos J.L. (1987). Analysis of epoxy resin curing kinetics using the Avrami theory of phase change. Polym. Eng. Sci..

[15] Ivankovic M., Incarnato L., Kenny J.M., Nicolais L. (2003). Curing kinetics and chemorheology of epoxy/anhydride system. J. Appl. Polym. Sci..

[16] Cheng K.C., Chiu W.Y., Hsieh K.H., Ma C.C.M. (1994). Chemorheology of epoxy resin Part I epoxy resin cured with tertiary amine. J. Mater. Sci..

[17] Bogetti T.A., Gillespie J.W. (1992). Process-Induced Stress and Deformation in Thick-Section Thermoset Composite Laminates. J. Compos. Mater..

[18] Kravchenko O.G., Kravchenko S.G., Pipes R.B. (2016). Chemical and thermal shrinkage in thermosetting prepreg. Compos. Part A Appl. Sci. Manuf..

[19] Kravchenko O.G., Kravchenko S.G., Pipes R.B. (2017). Cure history dependence of residual deformation in a thermosetting laminate. Compos. Part A Appl. Sci. Manuf..

[20] Madhukar M.S., Genidy M.S., Russell J.D. (2000). A New Method to Reduce Cure-Induced Stresses in Thermoset Polymer Composites, Part I: Test Method. J. Compos. Mater..

[21] Morgan R.J., O’Neal J.E. (1977). The microscopic failure processes and their relation to the structure of amine-cured bisphenol-A-diglycidyl ether epoxies. J. Mater. Sci..

[22] Vanlandingham M.R., Eduljee R.F., Gillespie J.W. (1999). Relationships between Stoichiometry, Microstructure, and Properties for Amine-Cured Epoxies. J. Appl. Polym. Sci..

[23] Cuthrell R.E. (1968). Epoxy polymers. II. Macrostructure. J. Appl. Polym. Sci..

[24] Vanlandingham M.R., Eduljee R.F., Gillespie J.W. (1999). Moisture diffusion in epoxy systems. J. Appl. Polym. Sci..

[25] Palmese G.R., McCullough R.L. (1992). Effect of epoxy-amine stoichiometry on cured resin material properties. J. Appl. Polym. Sci..

[26] Palmese G.R., McCullough R.L. (1994). Kinetic and thermodynamic considerations regarding interphase formation in thermosetting composite systems. J. Adhes..

[27] Hale A., Macosko C.W., Bair H.E. (1991). Glass transition temperature as a function of conversion in thermosetting polymers. Macromolecules.

[28] Gillham J.K. (1986). Formation and Properties of Thermosetting and High *T_g_* Polymeric Materials. Polym. Eng. Sci..

[29] López J., Ramírez C., Torres A., Abad M.J., Barral L., Cano J., Díez F.J. (2002). Isothermal curing by dynamic mechanical analysis of three epoxy resin systems: Gelation and vitrification. J. Appl. Polym. Sci..

[30] Lange J., Altmann N., Kelly C.T., Halley P.J. (2000). Understanding vitrification during cure of epoxy resins using dynamic scanning calorimetry and rheological techniques. Polymer.

[31] Chiou P.-L., Letton A. (1992). Modelling the chemorheology of an epoxy resin system exhibiting complex curing behaviour. Polym. Eng. Sci..

[32] Vyazovkin S., Sbirrazzuoli N. (1996). Mechanism and Kinetics of Epoxy-Amine Cure Studied by Differential Scanning Calorimetry. Macromolecules.

[33] Yue L., Maiorana A., Patel A., Gross R., Manas-Zloczower I. (2017). A sustainable alternative to current epoxy resin matrices for vacuum infusion molding. Compos. Part A Appl. Sci. Manuf..

[34] Nightingale C., Day R.J. (2002). Flexural and interlaminar shear strength properties of carbon fibre/epoxy composites cured thermally and with microwave radiation. Compos. Part A Appl. Sci. Manuf..

