# Supplementary material for: Effect of Curing Rate on the Microstructure and Macroscopic Properties of Epoxy Fiberglass Composites
Source: Polymers (Basel). 2018 Jan 27;10(2):125. doi: 10.3390/polym10020125 (PMC6415024; doi:10.3390/polym10020125)
Supplement: Supplementary file 1 [file polymers-10-00125-s001.docx]

Supporting Information


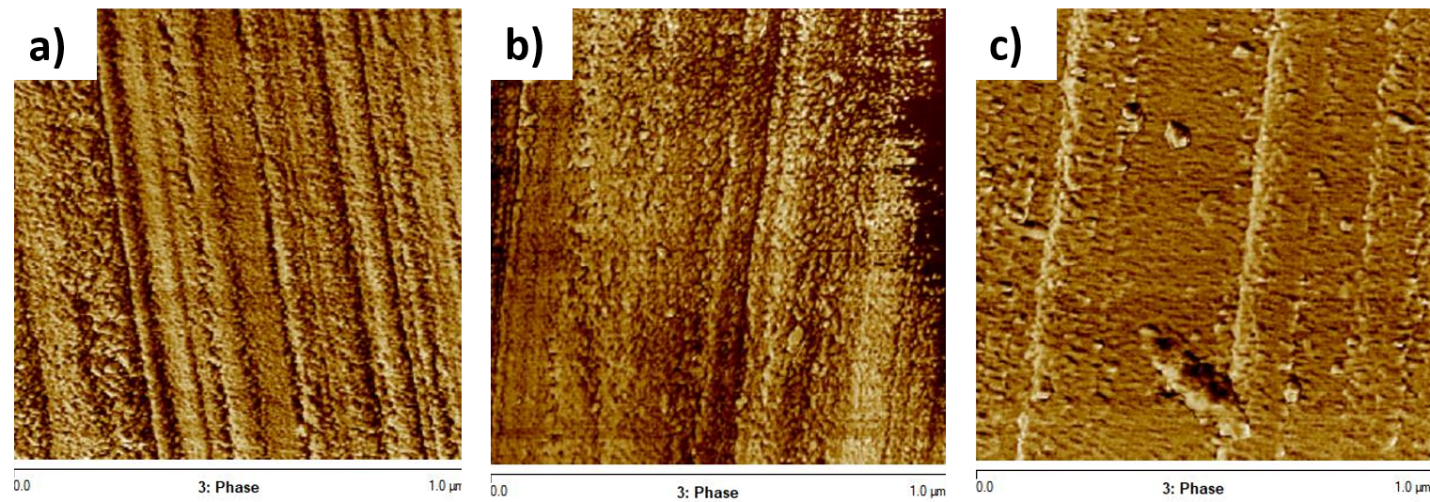


**Figure S1.** Phase images of (a) RT sample (b) 70 sample (c) 140 sample at 1μm scale size.


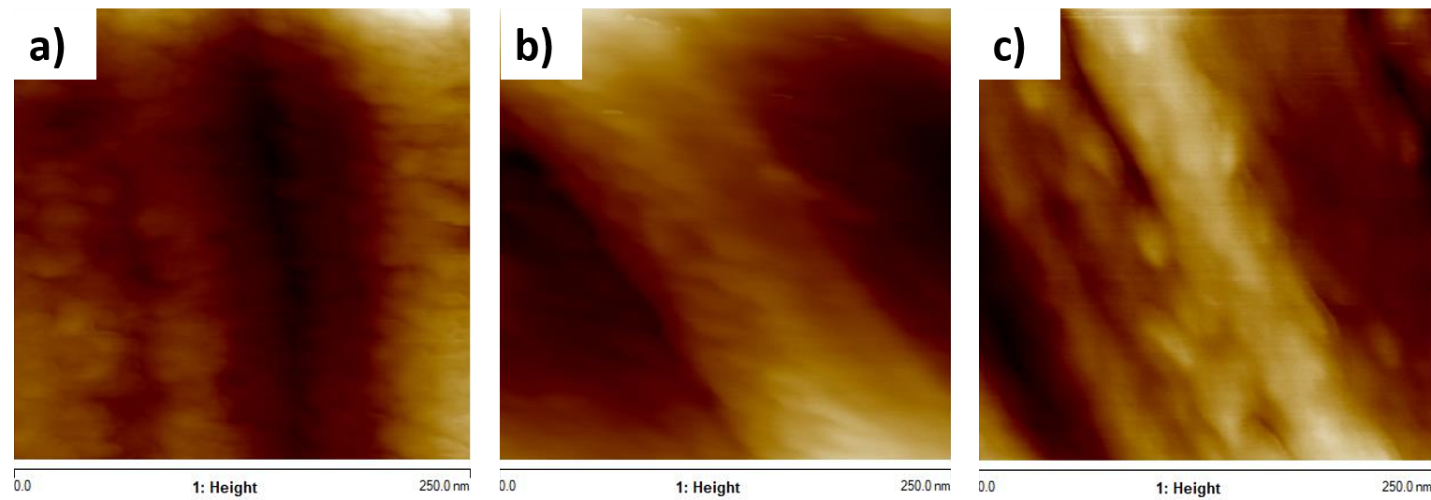


**Figure S2.** Height images of a) RT sample b) 70 sample c) 140 sample at 250nm scale size

**Table S1.** Percentage area of softer phase interspersed between the hard epoxy network

| **Sample** | **Dark spot percentage area (%)** |
| --- | --- |
| RT sample | 14.7 |
| 70 sample | 22.68 |
| 140 sample | 31.75 |

**Table S2.** Onset of thermal degradation at 5 weight % loss and peak degradation temperature for the samples cured at different rates

| **Sample** | ***T*_d5%_ (°C)** | ***T*_peak_ (°C)** |
| --- | --- | --- |
| RT sample | 345 | 361 |
| 70 sample | 348 | 362 |
| 140 sample | 350 | 359 |

**
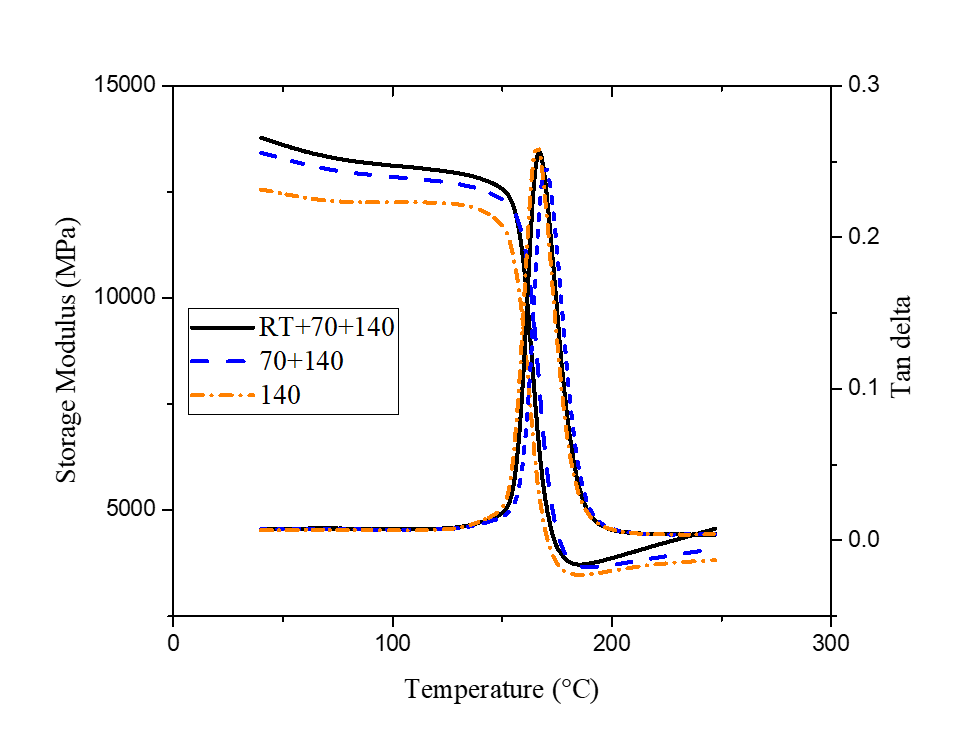
**

**Figure S3.** DMA curves for epoxy glass fiber composites

**
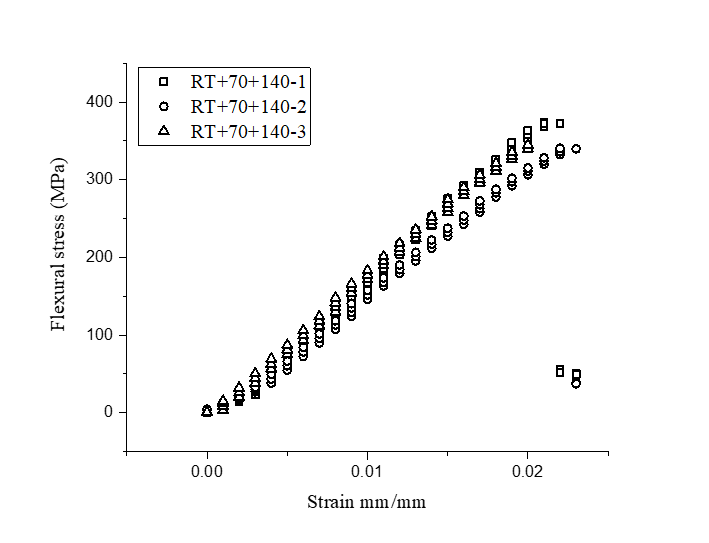
**

**Figure S4.** Flexural 3 point bending curves for RT sample

**
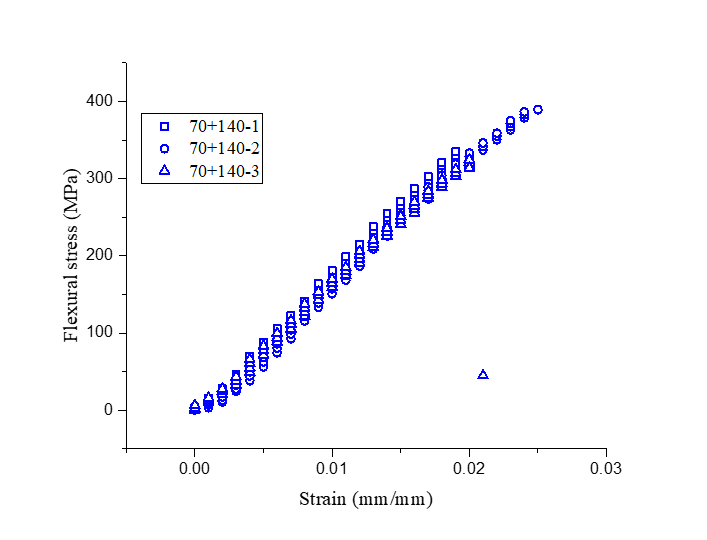
**

**Figure S5.** Flexural 3 point bending curves for 70 sample

**
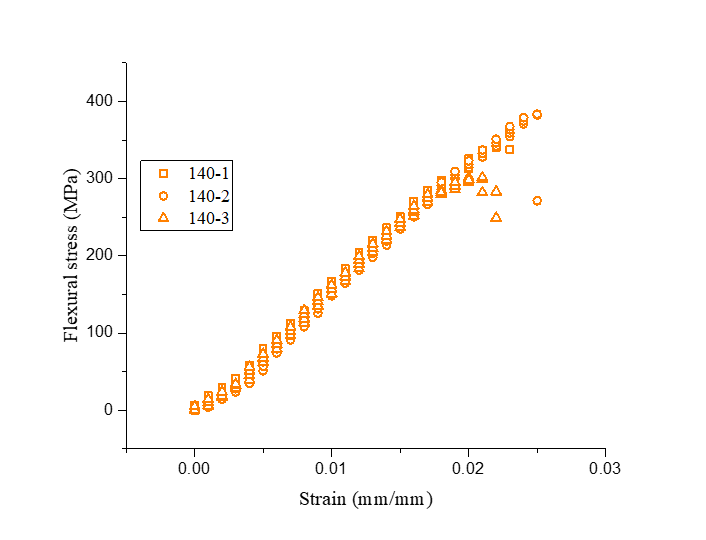
**

**Figure S6.** Flexural 3 point bending curves for 140 sample

**Table S3.** Dynamic Mechanical Analysis of sample without glass fiber mats.

| **Sample** | **Storage modulus at**  **40 °C (MPa)** | **Storage modulus at 200 °C (MPa)** | **Tg**  **(°C)** |
| --- | --- | --- | --- |
| RT sample | 2158 ± 18 | 9.1 ± 0.5 | 161 |
| 70 sample | 2165 ± 30 | 8.8 ± 0.4 | 159 |
| 140 sample | 2134 ± 52 | 7.2 ± 1.1 | 159 |

**
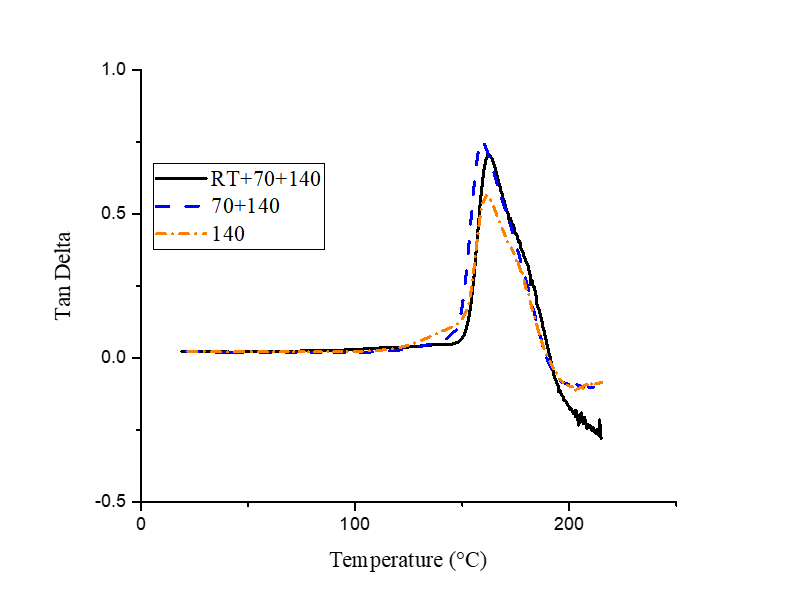
**

**Figure S7.** Tan delta vs temperature for the 3 curing cycles without glass fiber mats
